# Achieving Quality Integrated Care for Adolescent Depression: A Scoping Review

**DOI:** 10.1177/21501319221131684

**Published:** 2022-11-07

**Authors:** Diana Sarakbi, Dianne Groll, Joan Tranmer, Kim Sears

**Affiliations:** 1 Queen’s University, Kingston, ON, Canada

**Keywords:** primary care, depression, quality improvement, children, behavioral health

## Abstract

**Introduction:**

While primary care is often the first point of contact for adolescents with depression, more than half of depressed adolescents are either untreated or undertreated. A scoping review had been completed to summarize approaches for achieving quality integrated care in primary care focused on adolescent depression.

**Methods:**

The scoping review followed the methodological framework for scoping studies from Arksey and O’Malley. Articles were grouped into themes and mapped to 6 quality domains for integrated care from the practice integration profile survey and 3 levels of stakeholders based on WHO’s definition for health systems (patient/family, primary care team, and national/sub-national health system).

**Results:**

A total of 868 records were screened resulting in 22 articles at the patient/family-level (5/22), the primary care team-level (18/22), and the national/sub-national health system-level (16/22). The results highlighted multilevel approaches to support the delivery of quality integrated care for adolescent depression in primary care: (1) population-focused using patient registries, routine screening based on standardized algorithms, and patient-centered strategies, (2) team-driven where primary care clinicians collaborate with mental health clinicians as part of a primary care team, (3) evidence-based delivery of mental health services across the integrated care pathway from screening to follow-up visits, and (4) measurement-guided by leveraging the electronic health record infrastructure to learn from patient outcomes.

**Conclusion:**

More research is needed on how to provide quality integrated care for adolescent depression, specifically on patient engagement and retention, grounded in the frontline experiences of patients, families, and clinicians and supported by national and/or sub-national guidelines. A learning system could help integrate mental health services in primary care in a way that is consistent across the national and/or sub-national health system.

## Introduction

Depression was identified globally as one of the leading causes of illness and disability for adolescents by the World Health Organization (WHO).^1^ A 10-year longitudinal study examining the development of depression from pre-adolescence to young adulthood found the incidence of depression increases during adolescence, with approximately 14% of males and 28% of females experiencing a major depressive episode by the time they turn 18 years old.^2^ The COVID-19 pandemic further exacerbated this problem as social isolation and loneliness increased the risk of depression in adolescents.^3^ Adolescence is a critical period to screen for symptoms of depression because left untreated it may lead to negative long-term outcomes in adulthood at a higher cost to society, including impaired psychosocial functioning, and loss of productivity and income.^4-7^

Primary care is often the first point of contact for adolescents with depression.^8^ While primary care clinicians are more likely to screen patients with visible symptoms, they may have lower detection rates for internalized disorders like depression where patients tend to not report their symptoms.^9^ It is estimated that primary care clinicians identify symptoms of depression in less than half of presenting patients, and 1 in 5 adolescents receives the required dosage and treatment duration for antidepressant medications and 1 in 6 for psychotherapy sessions.^10^

Integrating mental health services in primary care, referred to as integrated care, promotes close collaboration between mental health and primary care clinicians to provide coordinated services to patients that includes screening, assessment, diagnosis, and treatment.^11,12^ Understanding how to deliver quality integrated care in primary care could help improve the identification and treatment of adolescents with depression.^13^

A review of the literature had been completed to summarize the existing evidence for delivering quality integrated care in primary care focused on adolescent depression. The concept of quality was guided by the 6 quality domains for integrated care from the Practice Integration Profile (PIP) survey to achieve better patient experiences and outcomes: (1) routine screening to identify cases, (2) consistent workflow for assessing, diagnosing, and treating patients, (3) comprehensive clinical services including non-pharmacological treatment options, (4) collaborative workspace, (5) ongoing communication and shared decision-making, and (6) patient engagement and retention strategies.^12^ Adolescence was described as the period of development between puberty and adulthood that generally corresponds to people aged 10 to 19 years old.^14^

## Methods

The scoping review consisted of 4 stages based on the methodological framework for scoping studies from Arksey and O’Malley^15^: (1) developing the search question and objectives, (2) identifying the inclusion criteria, screening, and selecting relevant studies, (3) extracting and analyzing data from selected articles, and (4) summarizing and reporting the findings.

### Search Question

The following central question was explored in the literature: Which approaches could contribute to the quality of mental health services in primary care for adolescents with depression?

### Search Strategy

The search strategy was developed in consultation with a library scientist from Queen’s University to ensure relevant terms and databases were selected based on the search questions. As recommended by the Joanna Briggs Institute for scoping reviews, the PCC (Population, Concept, and Context) framework was used to define the search terms based on the objectives of the literature review and search questions.^16^ This was an iterative process that included an exploratory search for articles using free-text terms to develop, pilot, and refine the search strategy. The PCC framework was also used to define the inclusion criteria for selecting relevant articles (Table 1).

**Table 1.** Literature Review Search Strategy.

Objectives	Inclusion criteria	Search terms
Quality integrated care in primary care for adolescents with depression	P (Population) Depression in adolescents generally between 10 and 19 years old	P (Population) Children/Child*/Adolescent(s)/Youth Depression/Depressive Disorder
	C (Concept) Providing quality mental health services across the integrated care pathway (from screening to follow-up visits)	C (Concept) Quality Collaborative/Integrated Care
	C (Context) Primary care setting Recent publications 2010 to 2021 Any country/language	C (Context) Primary Care/Primary Health Care Family Physician/Medicine General/Family Practice

* Conservative search that included “children/child” given the various age ranges in the literature.

Four databases were searched on January 3rd, 2022 (MEDLINE, APA PsycInfo, CINAHL, and Embase) using the search terms in the PCC framework as follows: (“Children” OR “Child” OR “Adolescent” Or “Adolescents” OR “Youth”) + (“Depression” OR “Depressive Disorder”)+ (“Quality” OR “Collaborative Care” OR “Integrated Care”) + (“Primary Care” OR “Primary Health Care” OR “Family Physician” OR “Family Medicine” OR “General Practice” OR “Family Practice”). A conservative approach was used by including the terms “child/children” in addition to “adolescent/youth” to capture articles focused on people between the ages of 10 and 19 given the variability in the age ranges for adolescence in the literature.

The default settings were used to include recent citations in all languages between January 1st, 2010 and December 31st, 2021 with the search terms mapped to “subject heading.” The search results were limited to recent articles published within the last 10 years to review up-to-date research on quality integrated, including its local context (eg, national strategy, and evidence-based guidelines).

### Study Selection

**Figure 1.**
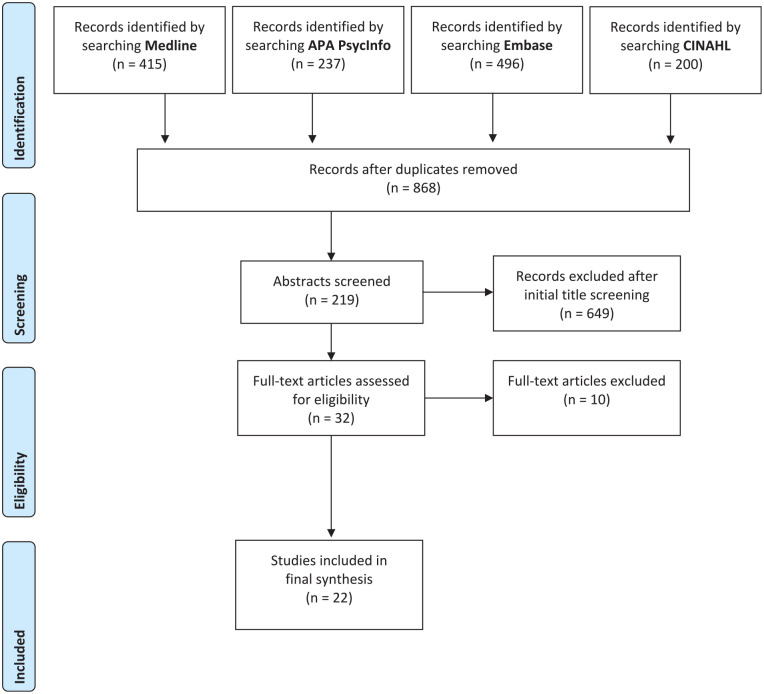
PRISMA study flow diagram.

The search and screening results were reported using the Preferred Reporting Items for Systematic Reviews and Meta-Analysis (PRISMA) study flow diagram (Figure 1).^17^ The search strategy resulted in 868 records after the duplicates were removed. The articles were screened by 2 reviewers using a 2-step process based on the following inclusion criteria: (1) primary research publications or literature reviews addressing one or more components of the integrated care pathway for depression (screening, assessment, diagnosis, treatment, and follow-up), (2) focused primarily on adolescents, and (3) in the context of the primary care setting.

The 2 reviewers screened the articles by titles and abstracts according to the inclusion criteria where 32 articles were retained for a full-text review with any disagreements resolved by consensus. Of the 32 articles, 10 were excluded based on study type (eg, editorials/commentaries, symposium summaries, and book chapters), and focus (eg, adult case study). The citations of excluded articles during the full-text review are referenced in Supplement 1. A total of 22 articles were included in the final synthesis.

### Analysis

The characteristics of each study were extracted and charted in a data extraction table by 2 reviewers that identified the following items: (1) title, authors, publication year, country, and context (national/sub-national policies, guidelines, and/or recommendations), (2) description of integrated care model, and (3) study design, sample size, aim, measures, and key findings (Supplement 2).

A thematic analysis was completed where the 22 retained articles were grouped into 12 themes using an inductive approach and mapped to the quality domains for integrated care from the PIP survey (case identification, workflow, clinical services, workspace, shared care, and patient engagement), and the 3 main levels of stakeholders based on WHO’s definition for health systems: the patient/family (service users), the primary care team (service providers), and the national/sub-national health system (government organizations/agencies).^12,18^

## Results

Of the 22 articles retained for the final synthesis, 18 of them were from the United States (US) (82%), 2 from Australia (9%), 1 from Chile (4%), and 1 was an international systematic review (4%). All the articles were published in English. There was 1 qualitative study (4%), 3 quality improvement initiatives (14%), 5 cross-sectional studies (23%), 5 retrospective cohort studies (23%), 7 randomized controlled trials (32%), and 1 systematic review (4%). The articles were mapped to the 6 quality domains for integrated care and the 3 levels of stakeholders where some articles covered more than 1 group: 5 articles at the patient/family-level, 18 articles at the primary care team-level, and 16 articles at the national/sub-national health system-level (Table 2).

**Table 2.** Organizing Framework for the Literature Review Results.

Stakeholder level	National/sub-national health system	Primary care team	Patient/family
Quality domain			
Case identification	■ Routine screening	■ Screening	■ Patient/family-clinician relationship ■ Parental involvement ■ Shared decision-making
Workflow	■ National strategy ■ Integrated care model ■ Evidence-based guidelines ■ Clinician training	■ Assessment and diagnosis ■ Treatment and follow-up	
Clinical services			
Workspace			
Shared care			
Patient engagement and retention	■ Health equity	■ Patient/family-clinician relationship	

### Patient/Family-Level

Five of the 22 articles addressed patient and/or family-related themes contributing to all the quality domains for integrated care for adolescent depression from screening to follow-up: patient/family-clinician relationship, parental involvement, and shared decision-making.

#### Patient/family-clinician relationship

In one qualitative study, adolescents with depression identified themes that contributed to a positive patient experience, including the characteristics of a “youth-friendly” clinician (understanding, respectful, unbiased, and proactive with follow-up appointments), choice of treatment (psychotherapy sessions and/or antidepressant medications), and ongoing patient-clinician communication to solve problems including the opportunity to change clinicians in cases of lack of engagement or break-down in the relationship.^19^

A randomized controlled trial comparing a remote collaborative intervention for adolescent depression to usual care found that a positive patient experience was associated with better outcomes. The intervention group (n = 65) reported greater satisfaction with the services received compared to the control group (n = 78) (Wilcoxon rank sum test *P* = .04), and satisfaction with the services received was correlated with a greater decrease in depressive symptoms after 12 weeks for both groups (β = −4.3, 95% CI [−7.2, −1.3]).^20^

#### Parental involvement

One randomized controlled trial reported benefits of involving parents of adolescent patients in their treatment. They found that parental involvement resulted in greater adherence and improved patient outcomes where 86% (43/50) of patients in the intervention group met treatment standards for medications and/or psychotherapy compared to 32% (68/211) of patients where parents were not involved as part of the overall treatment plan in a similar referenced study.^21,22^ Another randomized controlled trial (n = 207) noted an age-related decline in the use of mental health services where older adolescents (within the 13-21 age range) had lower treatment rates (*P* < .0001). The authors indicated this finding could be due to a decrease in parental involvement in treatment plans as adolescents get older.^23^

#### Shared decision-making

One cross-sectional study asked 57 primary care clinicians to identify the patient/family characteristics that influence their decision-making process about treatments for depression. Most of the clinicians reported quality of family support (n = 46 or 81%) and more than half identified parent’s understanding of depression as contributing factors (n = 36 or 63%). Although considering patient and parental preferences can improve treatment adherence, less than half of the clinicians identified shared decision-making as an important factor that influenced their choices of treatment for depression with 40% (n = 23) selecting “parent’s preference for treatment” and 42% (n = 24) for “adolescent’s preference for treatment.”^24^

### Primary Care Team-Level

Eighteen of the 22 articles (82%) covered themes at the primary care team-level contributing to all the quality domains for integrated care within the context of adolescent depression: screening, assessment, diagnosis, treatment, and/or follow-up.

#### Screening, assessment, and diagnosis

A systematic review on screening for depression in children and adolescents found that the Beck Depression Inventory (BDI) and the Patient Health Questionnaire for Adolescents (PHQ-A) were accurate in identifying adolescents with depression in primary care based on the limited evidence available in the literature. The BDI and PHQ-A reported the highest sensitivity (73%-90%) and specificity (81%-94%) compared to other screening instruments.^25^

Nine of the 22 articles evaluated the benefits of screening to detect adolescent depression in primary care where 5 articles used the PHQ-A, 2 used a general mental health screening tool (Pediatric Symptom Checklist), and 2 used the PHQ-A in combination with other assessment tools.

The PHQ-A showed a high adherence rate with 76% (6981/9149) of adolescent patients screened at their annual visit at age 16 in a cross-sectional study.^26^ The PHQ-A also increased the number of adolescent patients screened for depression from 34% to 97% over the 7-month period of a quality improvement collaborative. Adolescents from the quality improvement group (n = 792) were 37.5 times more likely to be screened with a validated tool than adolescents in the control group receiving usual care (n = 772) (95% CI [7.67, 183.48] *P* < .0005).^27^ Another quality improvement project found similar results over a 2-month period where 75% (73/98) of adolescent patients received a documented depression screen compared to none during the same 2-month period from the previous year.^28^ One retrospective cohort study found that having an integrated screening system in the Electronic Health Record (EHR) increased screening rates where 79% (15 842/20 053) of adolescent patients were screened with the PHQ-A compared to only 7% (2333/32 495) within the 12 months before the intervention.^29^

Three studies looked at the relationship between screening and percentage of adolescent patients diagnosed with depression. A retrospective cohort study found that screening using the Pediatric Symptom Checklist increased the odds of patients diagnosed with depression in primary care clinics with an integrated care model where primary care clinicians collaborate with mental health clinicians to deliver services (n = 13 572) compared to clinics with mental health screenings only (n = 15 300) (OR = 2.03; 95% CI [1.58, 2.59] *P* < .001).^30^ However, the overall percentage of patients diagnosed with depression was relatively low at 3% compared to the 20% of adolescents with depression in community samples.^31^ Using a depression-specific assessment tool like the PHQ-A may help improve the detection of depression symptoms. A quality improvement project using the PHQ-A increased the number of adolescents diagnosed with depression from 5% (15/282) to 17% (15/88).^5^ Another quality improvement project also using the PHQ-A found a 13.3% increase in the rate of new depression diagnoses (*P* = .0017) in the pre (n = 86) and post (n = 98) implementation samples.^28^

Two studies examined the relationship between screening and treatment. A retrospective cohort study found patients who scored positively during screening were about 9 times more likely to receive treatment than patients who screened negative (24.3% vs 2.6%, χ^2^ = 59.65, *P* < .001).^32^ Another cross-sectional study found that 88% (130/148) of adolescent patients who were diagnosed with depression received treatment within the primary care clinic in the form of psychotherapy and/or medication.^33^

All 9 studies evaluating the benefits of screening found positive results primarily with the PHQ-A and recommended completing annual screening during well visits to assess both the physical and mental health needs of adolescents. A quality improvement project found that of the 77 patients screened with the PHQ-A, more patients showed symptoms of depression and were screened during sick visits (57%, 44) than well visits (43%, 33), and recommended also screening for depression during sick visits.^5^

#### Treatment effectiveness

Six of the 22 studies studied the effectiveness of treatments for adolescent depression which consisted of 1 systematic review, 4 randomized controlled trials, and 1 retrospective cohort study. The treatments that were tested were Cognitive Behavioral Therapy (CBT), Interpersonal Psychotherapy (IPT), and antidepressant medications. The systematic review found treatment options including fluoxetine, combined fluoxetine and CBT, and escitalopram showed benefits among adolescents, with no associated harms based on the limited evidence available.^25^

A randomized controlled trial evaluated the effectiveness of IPT with antidepressant medication as needed compared to enhanced treatment as usual of referral to mental health services. Trained primary care clinicians provided IPT with ongoing supervision by a mental health clinician. About half of the adolescent patients (15/29) improved after 8 weeks of brief IPT sessions and did not need medication nor referral to specialized mental health services. The depression symptoms of patients (Children’s Depression Rating Scale [CDRS-R] Cohen’s *d* = 0.35) and their overall severity (Clinical Global Impressions Scale [CGI-S] Cohen’s *d* = 0.84) improved more in the IPT group compared to the control group. At week 16, patients who received treatment in both groups experienced better outcomes on the CDRS-R and CGI-S (*P* < .05 for each). Therefore, patients in the IPT group benefited from earlier treatment and were able to alleviate their symptoms sooner as only 37% (7/19) of patients in the control group received treatment.^34^

Three studies evaluated CBT as a treatment option for depression and found that patients who received CBT responded to treatment and recovered earlier compared to the control group.^22,35,36^ Treatments were provided by either primary care or master-level clinicians trained in CBT with ongoing supervision by a mental health clinician.

In the randomized controlled trial, the control group (n = 106) had an average of 30 weeks to recovery (95% CI [25.3, 34.7]) compared with an average of 22.6 weeks for the CBT group (n = 106) (95% CI [18.7, 26.5]. After 1 year, the control group experienced higher rates of hospitalizations compared with the CBT group (8.5% vs 0.9%, *P* = .01). Therefore, providing brief CBT in primary care decreased the risk of recurrent major depressive episodes and the use of hospital services for adolescents.^35^ In a retrospective cohort study, the CBT group (n = 162) had better adjusted rates of depression remission (31% vs 20%, *P* = .035) and treatment response (44% vs 30%, *P* < .001) than the control group (n = 499).^36^ One randomized controlled trial had a more comprehensive approach where treatment options consisted of CBT, antidepressant medication and/or both. There were higher decreases in CDRS-R scores after 12 months in the CBT group (n = 50) with mean score of 27.5 (95% CI [23.8, 31.1]) compared with 34.6 (95% CI [30.6, 38.6]) in the control group (n = 51). The CBT group was more likely than the control group to achieve depression response (67.6% vs 38.6%, OR = 3.3, 95% CI [1.4, 8.2] *P* = .009) and remission (50.4% vs 20.7%, OR = 3.9, 95% CI [1.5, 10.6] *P* = .007) after 12 months.^22^

One randomized controlled trial looked at the effectiveness of medications using an integrated care model where primary care clinicians received remote guidance from mental health clinicians on diagnosis and treatment of adolescent depression. There were no significant differences in patient outcomes between the intervention (n = 65) and control groups (n = 78) after 12 weeks. This may be explained by the lower adherence to treatment due to a high turnover rate of primary care clinicians in remote areas.^20^

#### Treatment adherence and follow-up

Three of the 22 studies evaluated adherence to treatment as one of their measures. In one retrospective cohort study, only 11% (42/137) of adolescent patients received medication treatment, and in a randomized controlled trial, only a third of the adolescent patients (44/143) took their medications as prescribed.^20,29^ Low adherence to treatments could be explained by a high turnover rate of primary care clinicians in remote areas and difficulties training new clinicians.^20^ A randomized controlled trial with a high adherence to treatment rate (86%, 43/50) identified proactive efforts for following-up with the adolescent patient as one of the contributing factors.^22^

Two studies examined follow-up rates. A cross-sectional study found that out of 130 adolescent patients only 55% (n = 71) had at least 1 follow-up visit, 22% (n = 29) had at least 2 follow-up visits, and 12% (n = 15) had 3 or more follow-up visits within 12 weeks.^33^ These low rates highlight the need for a more proactive approach to following-up with patients as part of their relapse prevention plan. Another cross-sectional study showed that having an automated system in the EHR helped increase follow-up rates where 75% (n = 349/463) of patients had a follow-up visit within 1 year.^26^

### National/Sub-National Health System-Level

Sixteen of the 22 articles (73%) described the context of their study. Themes were identified at the national/sub-national health system-level to support the quality domains for integrated care focused on adolescent depression: (1) routine screening strategy to facilitate case identification, (2) national strategy, integrated care model, evidence-based guidelines, and clinician training activities to support the quality domains on workflow, clinical services, workspace, shared care and patient engagement and retention, and (3) health equity strategy to address barriers to patient engagement and retention specifically within minority populations.

#### National strategy

The 2 studies completed in Australia described their national strategies to improve the quality of mental health services in primary care including the National Mental Health Strategy (1992), a 5-year mental health plan (1993-2014), a National Action Plan of Mental Health under the Council of Australian Governments (2006-2012), the Better Access initiative (2006), and annual National Report Cards on Mental Health and Suicide Prevention (2012).^37^ A publicly funded integrated care model was developed in 2006 to provide mental health services in primary care for people 12 to 25 years old.^19^

#### Integrated care model

Eight of the articles provided a specific definition for integrated care referred to as either “collaborative care,” “integrated behavioral health,” or “integrated mental health services.” Supplement 3 references the definition provided in each of the 8 articles.^20,23,29,30,34,36,38,39^ The following common characteristics emerged for this health service delivery model with the aim of expanding the reach of mental health services to primary care: population-focus, stepped approach to care, team-driven, evidence-based, and measurement-guided. The components of an integrated care model that were linked to increased screening rates for adolescent depression were using evidence-based guidelines and training of clinicians.^39^

Two of 3 CBT studies referenced in this scoping review were analyzed for cost-effectiveness to determine the value of treating adolescent depression in primary care compared to usual care.^22,35^ These studies evaluated the costs of integrated care for CBT treatments and found it to be a cost-effective method for treating adolescent depression in primary care. In the first study, the CBT group (n = 50) received evidence-based treatment on-site with regular follow-up while the control group (n = 51) received screening results for depression with a recommendation for further assessment and treatment as applicable. The study found no significant differences in costs between the CBT ($5161; 95% CI [$3564, $7070]) and control ($5752; 95% CI [$3814, $7952]) groups. The mean incremental cost-effectiveness ratio was $18 239 (95% CI, dominant to $24 408) per Quality-Adjusted Life Year (QALY) gained where CBT treatment resulted in cost savings and increase in QALYs.^38^ The second study found that the CBT group had on average 26.8 more Depression-Free Days (DFDs) (*P* = .044) and .067 more QALYs (*P* = .044) compared with the control group providing treatment as usual after 1 year. The costs were $4976 less (*P* = .025) in the CBT group than the control group after 2 years.^40^

#### Strategy for routine screening

Nine articles referenced the US Preventive Services Task Force guidelines recommended in 2009 to screen adolescents 12 to 18 years old for symptoms of depression in primary care if appropriate mental health services were available for this population in this setting.^22,26-29,32-34,36^ In 2016, the American Academy of Pediatrics (AMP) recommended screening for depression every year once adolescents turn 11 years old.^5^ This recommendation was supported by primary care physicians in a 2004 AMP survey where 80% of pediatricians stated that they were responsible for screening for mental disorders.^27^ Although AMP developed training materials to support routine screening of adolescents for depression in primary care, an AMP survey completed in 2013 found that screening rates in primary care remained low at less than 25%.^5^ Whereas, one cross-sectional study explained that in the US state of Minnesota screening adolescents 12 to 20 years old for mental disorders and/or specifically depression is mandatory during well child visits with monthly reporting of screening rates, and reported a mean screening rate of 87% (SD = 12.62%) for adolescent depression.^39^

#### Evidence-based guidelines for treatment

The National Guidelines for Adolescent Depression in Primary Care recommended CBT and/or antidepressant medications for depression.^24^ However, in 2004, the US Food and Drug Administration cautioned the use of antidepressants in children and adolescents because of the potential increased risk of suicide.^35^ While there are many recommendations available to support the management of adolescent depression, few are based on evidence. Clinician training is needed to identify and adhere to evidence-based guidelines for screening and managing adolescent depression in primary care.^41^

#### Clinician training

Two cross-sectional studies evaluated clinician adherence to evidence-based guidelines for depression. The first study found the lowest compliance was reported for primary care clinicians compared to other health settings with low adherence to assessment bundles for depression at 30% (95% CI [11.7, 55.3] and management bundles for depression at 32% (95% CI [7.7-66.5]). These results provided baseline benchmarks to improve adherence through clinician training and the use of automated reminders in the EHR platform.^37^ The second study found that only a third of 58 clinicians recommended an antidepressant medication (25% for moderate symptoms and 32% for severe symptoms). Primary care clinicians who were knowledgeable in antidepressants were more likely to prescribe medications for depression (OR = 1.72 [95% CI 1.14, 2.59] *P* = .009) and have access to an onsite mental health clinician (OR = 5.13 [95% CI 1.24, 21.2] *P* = .02). Factors that influenced the clinician’s treatment choices included knowledge of depression and evidence-based treatments, level of comfort with managing psychosocial problems, and availability of a mental health clinician within the primary care clinic.^24^

#### Health equity

One article explained that the National Network of Child Psychiatry Access Programs provided mental health services in primary care, but the availability of these services depended on location.^36^ In Chile, the Chilean Ministry of Health issued recommendations for treating adolescent depression in primary care and developed a plan to scale mental health services to include clinics with limited resources.^20^ Two articles compared the racial differences in treatment uptake. In the retrospective cohort study of 956 patients, Hispanics (n = 548) and Blacks (n = 83) were more likely to receive lower quality mental health services for depression compared to White patients (n = 298). Minority patients were approximately 30% less likely to receive adequate treatment for depression (Hispanics OR = 0.67; 95% CI [0.6, 0.8]) (Blacks OR = 0.66; 95% CI [0.6, 0.8]).^42^ The randomized controlled trial found treatment rates were higher in the CBT group (n = 211) when English was the primary language spoken at home (67%, 141), but there were no differences for other languages (*P* = .023). Future research is needed to better understand the barriers to treatment uptake among minority adolescents in primary care.^23^

## Discussion

The literature review results highlighted several multilevel approaches to support the delivery of quality integrated care for adolescent depression in primary care.

Integrated care had been incentivized in the US to help achieve the triple aim of improving patient experiences and outcomes while reducing costs.^23^ Restructuring funding models to support the delivery of integrated health services focused on patient needs is a foundational national/sub-national strategy to facilitate quality integrated care at the primary care team-level, specifically the quality domains on collaborative workspace, ongoing communication, and shared decision-making. It is recommended to have dedicated funding for mental health services in primary care and explore value-based payment models to incentivize collaboration between primary care and mental health clinicians that is focused on adherence to evidence-based guidelines, patient experience, and outcomes.^43^

Multilevel approaches are needed to support the quality domain for integrated care focused on screening adolescents for symptoms of depression. Having a policy for routine screening for adolescent depression during annual well visits, programming automated reminders in the EHR platform, and using evidence-based guidelines with clinician training are associated with increased screening rates.^29,32,39^ Routine screening is especially important for mental disorders characterized by internalized symptoms like depression.^9^ Using a validated assessment instrument specific to depression like the PHQ-A is associated with higher detection rates of depression.^5,25-28^

Multilevel approaches are also needed to consistently assess, diagnose, and treat adolescents with depression and provide comprehensive mental health services including non-pharmacological treatment options as recommended by the quality domains on workflow and clinical services. Having a comprehensive approach to treating depression in primary care that includes CBT, IPT, and/or antidepressant medications combined with clinician training and on-site presence of mental health clinicians may contribute to better patient experiences and outcomes.^22,25,34-36^ CBT was specifically associated with a decrease in the use of hospital services and was a cost-effective treatment method for adolescent depression in primary care.^35,38,40^ Another quality domain to consider as part of the treatment plan for adolescent depression is patient engagement and retention strategies. Factors contributing to treatment adherence included patient-clinician relationship and parental involvement, shared decision-making, and ongoing communication supported by automated reminders in the EHR for follow-up visits.^19,20,22,26^ More research is needed to better understand the barriers to treatment uptake amongst adolescence, specifically within minority populations to support health equity.^23,42^

An evidence-based approach is needed for implementing the quality domains for integrated care in a way that is consistent across the national and/or sub-national health system.^44^ It’s recommended to consider the following multilevel approaches when developing an integrated care model for mental health services in primary care focused on adolescent depression: (1) population-focused using patient registries, routine screening based on standardized algorithms, and patient-centered strategies (eg, shared decision-making, active follow-up to support treatment adherence, and stepped approach to care based on patient’s response to treatment), (2) team-driven where primary care clinicians (eg, pediatricians and nurses) collaborate with mental health clinicians (eg, psychologists and social workers) as part of a primary care team, (3) evidence-based delivery of mental health services across the integrated care pathway from screening to follow-up visits, and (4) measurement-guided by leveraging the EHR infrastructure to learn from patient outcomes including any behavioral side effects from the use of antidepressants.^43-45^

A learning system could leverage real world evidence based on the frontline experiences of patients, families, and clinicians to continuously learn how to achieve the quality domains for integrated care focused on adolescent depression and inform supportive national/sub-national policies/strategies based on local context.^46^

## Conclusion

Providing quality mental health services in primary care for adolescents with depression is a collaborative effort between policy makers, primary care and mental health clinicians, and patients and their families. A learning system could help integrate mental health services in primary care in a way that is consistent across the national and/or sub-national health system. More research is needed on how to provide quality integrated care for adolescent depression, specifically to achieve the quality domain on patient engagement and retention which includes following-up with patients as part of their relapse prevention plan and addressing barriers to treatment uptake with a focus on minority populations.

## Supplemental Material

sj-docx-1-jpc-10.1177_21501319221131684 – Supplemental material for Achieving Quality Integrated Care for Adolescent Depression: A Scoping Review Click here for additional data file. Supplemental material, sj-docx-1-jpc-10.1177_21501319221131684 for Achieving Quality Integrated Care for Adolescent Depression: A Scoping Review by Diana Sarakbi, Dianne Groll, Joan Tranmer and Kim Sears in Journal of Primary Care & Community Health

sj-docx-2-jpc-10.1177_21501319221131684 – Supplemental material for Achieving Quality Integrated Care for Adolescent Depression: A Scoping Review Click here for additional data file. Supplemental material, sj-docx-2-jpc-10.1177_21501319221131684 for Achieving Quality Integrated Care for Adolescent Depression: A Scoping Review by Diana Sarakbi, Dianne Groll, Joan Tranmer and Kim Sears in Journal of Primary Care & Community Health

sj-docx-3-jpc-10.1177_21501319221131684 – Supplemental material for Achieving Quality Integrated Care for Adolescent Depression: A Scoping Review Click here for additional data file. Supplemental material, sj-docx-3-jpc-10.1177_21501319221131684 for Achieving Quality Integrated Care for Adolescent Depression: A Scoping Review by Diana Sarakbi, Dianne Groll, Joan Tranmer and Kim Sears in Journal of Primary Care & Community Health
