# Supplementary material for: Achieving Quality Integrated Care for Adolescent Depression: A Scoping Review
Source: J Prim Care Community Health. 2022 Nov 7;13:21501319221131684. doi: 10.1177/21501319221131684 (PMC9647275; doi:10.1177/21501319221131684)
Supplement: sj-docx-1-jpc-10.1177_21501319221131684 – Supplemental material for Achieving Quality Integrated Care for Adolescent Depression: A Scoping Review [file sj-docx-1-jpc-10.1177_21501319221131684.docx]

Supplement 1. List of Excluded Studies

1. Courtney, D.B., et al., Quality appraisal of clinical practice guidelines for depression in children and adolescents. Journal of the American Academy of Child and Adolescent Psychiatry, 2016. 55(10 Supplement 1): p. S172.
2. Ginsburg, A.D., et al., An Examination of Screening Tools for Collaborative Care of Adolescent Depression. The Journal of clinical psychiatry, 2018. 79(4).
3. Hernandez, A.M., Increasing Provider Compliance with Adolescent Depression Screenings in A Primary Care Clinic. 2020, The University of Arizona.
4. Kopecky-Wenzel, M. and R. Frank, Primary care by general practitioners: Recognition of depression in children and adolescents. Monatsschrift fur Kinderheilkunde, 2010. 158(9): p. 836-842.
5. Lahey, R., et al., Identifying and managing depression through collaborative care: expanding social work's impact. Social Work in Health Care, 2019. 58(1): p. 93-107.
6. McCarthy, A.E., Screening and management of adolescent depression in a large pediatric care network. Dissertation Abstracts International: Section B: The Sciences and Engineering, 2020. 81(9-B): p. No-Specified.
7. Neto, M.L.R., et al., Depression in children and adolescents: Family narratives in Brazilian primary attention. HealthMED, 2012. 6(9): p. 2950-2954.
8. Radovic, A., et al., "She thought that the girl had just made it up": Primary care provider perceptions of parental barriers to adolescent depression care. Journal of Adolescent Health, 2014. 54(2 SUPPL. 1): p. S19-S20.
9. Radovic, A., E. Miller, and B. Stein, Why are pediatric primary care providers reluctant to prescribe antidepressants to teens? Clinical Practice, 2014. 11(4): p. 393-396.
10. Yucel, A., et al., Pathway to depression care in children and adolescents first identified by primary care providers versus psychiatrists. Journal of the American Academy of Child and Adolescent Psychiatry, 2016. 55(10 Supplement 1): p. S177.
