# Supplementary material for: Achieving Quality Integrated Care for Adolescent Depression: A Scoping Review
Source: J Prim Care Community Health. 2022 Nov 7;13:21501319221131684. doi: 10.1177/21501319221131684 (PMC9647275; doi:10.1177/21501319221131684)
Supplement: sj-docx-2-jpc-10.1177_21501319221131684 – Supplemental material for Achieving Quality Integrated Care for Adolescent Depression: A Scoping Review [file sj-docx-2-jpc-10.1177_21501319221131684.docx]

Supplement 2. Data Extraction Table of Included Studies

| **Author/Year/Title/**  **Country** | **Study Design** | **Context** | **Sample Size** | **Aim** | **Integrated Care Model** | **Measures** | **Key Findings** |
| --- | --- | --- | --- | --- | --- | --- | --- |
| Wright 2016 (U.S.)  The Costs and Cost-effectiveness of Collaborative Care for Adolescents With Depression in Primary Care Settings: A Randomized Clinical Trial. | Experimental Study, Randomised Controlled Trial. | None specified. | 101 patients randomised to the intervention (n = 50) and usual care (n = 51) groups from 9 primary care clinics in the Group Health system in Washington State. Patients were 13-17 years old with depression. | To evaluate the costs and cost-effectiveness of a collaborative care model for treatment of adolescent major depression in primary care. | The Reaching Out to Adolescents in Distress (ROAD) collaborative is a 12-month intervention, including an initial in-person session, delivery of evidence-based treatments, and regular follow-up by clinicians.   Patients in the usual care control group received depression screening results and could access mental health services and obtain medications through Group Health. | Intervention costs and per capita health plan costs calculated from the payer perspective using administrative records.   Primary effectiveness outcome was the difference in Quality-Adjusted Life-Years (QALYs) between groups from baseline to 12 months calculated using Child Depression Rating Scale's Revised scores.  Cost and QALYs were used to calculate an incremental cost-effectiveness ratio. | Health plan costs were not significantly different between the intervention ($5161; 95% CI [$3564, $7070]) and usual care ($5752; 95% CI [$3814, $7952]) groups.   Mean incremental cost-effectiveness ratio was $18 239 (95% CI, dominant to $24 408) per QALY gained, with dominant indicating that the intervention resulted in both a net cost savings and a net increase in QALYs.  The ROAD collaborative is cost-effective similar to adult collaborative intervention care and other pediatric interventions. |
| Buchanan 2020 (U.S.)  Integration of behavioral health services and adolescent depression screening in primary care. | Correlational, Cross-Sectional Study. | (1) Mandatory screening of adolescents 12-20 years old at well-child visits for general mental health and/or specifically depression.   (2) Monthly reporting of data, including adolescent screening rate.  Benchmark: In the US, average national rate for standardized behavioural/emotional screenings is about 20% in pediatric primary care. | 82 clinics within 13 organizations providing primary care services to adolescents. Clinicians (medical providers, behavioural health providers, and nurses) and administrators were required to complete one survey together per clinic. | To evaluate the relationship between level of integration of behavioural health services in primary care and adolescent depression screening rate. | Various levels of integration of behavioural health adopted by clinics across the state. | Site Self-Assessment (SSA): 18-item measure of patient/family-centered integrated care in clinics.  Adolescent depression screening tools (various): Patient Health Questionnaire, Pediatric Symptom Checklist, Kutcher Depression Scale, Beck Depression Inventory, Child Depression Inventory, and Global Appraisal of Individual Needs screens for mental health and substance abuse.  Benchmark: In the US, average national rate for standardized behavioural/emotional screenings is about 20% in pediatric primary care. | Mean screening rate of adolescent depression: 87.03% (SD=12.62%). Mean SSA score of 6.35/10 (SD=1.66).   While overall level of integration was not related to adolescent depression screening rate (B=0.10, SD=0.12, p=.41), several individual components of integration were positively/negatively associated with screening.  Use of evidence-based practices (B=0.38, SD=0.11, p=.001) and education/training (B=0.39, SD=0.12, p=.001) were positively associated with screening.   Organizational leadership for integrated care (B= -0.20, SD=0.10, p=.03), funding access (B= -0.44, SD=0.15, p=.002) and linking to community resources (B= -0.21, SD=0.10, p=.03) were negatively associated with screening. Higher screening rates could be linked to higher standards/expectations from clinic personnel, and availability of behavioural health expertise onsite.  Survey responses were based on the perception of clinic personnel. It's recommended to use a multilevel organizational framework for future research. |
| Pop 2019 (U.S.)  VitalSign6: Screening, diagnosis, and treatment of depression for adolescents presenting to pediatric primary and specialty care settings. | Correlational, Cross-Sectional Study. | The US Preventive Services Task force and the American Academy of Pediatrics recommends screening, diagnosis and treatment of depression by pediatric primary care clinicians. | Patients 10-17 years old from 25 primary care clinics screened for symptoms of depression (n=2,625) between Aug 2014-May 2018. Participating clinicians consisted of physicians (n=59), nurse practitioners (n=22) and physician assistants (n=13). | To evaluate the impact of VitalSign6, a program designed to support pediatric primary clinicians to screen, diagnose, and initiate depression treatment. | The VS6 software supports clinicians in the diagnosis and treatment plan based on an algorithm designed to consider symptoms, adherence, side effect burden, and time elapsed.   Clinician review self-report measures, clinical interview results, and decision support algorithm to detect and diagnose depression.   Training and consultation on depression and measurement-based care provided by the VS6 team, which included psychiatrists, psychologists and other mental health clinicians. | PHQ-2 (Patient Health Questionnaire-2) to screen for symptoms of depression. If negative, PHQ-2 administered again in 1 year. If positive, PHQ-9 is completed.  PHQ-9 to assess level of severity of depression.  Number of follow-up visits for patients diagnosed with depression. Benchmark was 3 visits within 12 weeks. | Most patients diagnosed with depression (88%, n=130) received treatment onsite (e.g., psychotherapy and/or medication).  Patients who received treatment, 54.6% (n=71) had at least 1 follow-up visit within 12 weeks, 22.3% (n=29) had at least 2 follow-up visits, and only 11.5% (n=15) had 3 or more follow-up visits.   Standardized screening methods can help clinicians detect and diagnose depression. However, the low follow-up rates suggest the need for a more proactive approach. |
| Mufson 2018 (U.S.)  Stepped care interpersonal psychotherapy treatment for depressed adolescents: A pilot study in pediatric clinics. | Experimental Study, Randomised Controlled Trial. | The U.S. Preventive Services Task Force guidelines recommended in 2009 expanding screening and detection of depression in primary care when treatment is available. | 48 adolescent patients (mean age 15.9 years old [SD = 2.2]), 30 parents/primary care givers of the patients, and 7 clinicians (6 pediatricians and 1 nurse practitioner) from urban pediatric primary care clinics serving primarily Latino youth. | To evaluate the impact of the Stepped Collaborative Care Treatment Model (SCIPT-A) for Adolescents with depression. | A Stepped Collaborative Care Treatment Model (SCIPT-A) for Adolescents with depression using a brief version of Interpersonal Psychotherapy for Adolescents (IPT-A) and antidepressant medication (as needed).   IPT-A training provided in a 1-day seminar with a treatment manual, and weekly group supervision, and in-service seminar on prescribing antidepressants with an initial medication consultation as well as ongoing consultation as needed.  Phase I: Initial dose of 8 treatment sessions. Phase II: Depending on symptoms, patient entered maintenance treatment of 3 IPT-A sessions delivered over the course of 8 weeks, OR continued with weekly IPT-A sessions with the addition of antidepressants (fluoxetine) with medication visits at weeks 1, 2 ,4, 6 and 8.  Enhanced Treatment as Usual (E-TAU) consisted of referral to services within the clinic or externally with regular calls by a psychologist to follow-up on treatment referrals. | Diagnosis of depression was based on Children's Depression Rating Scale, Revised (CDRS-R), administered to patient and parent.   Patient Health Questionnaire (PHQ-9) to assess severity of depression.  The Clinical Global Impression scale (CGI) to evaluate response to treatment.  Children's Global Assessment Scale (CGAS) to evaluate overall level of functioning.   Social Adjustment Scale-Self Report (SAS-SR) to assess social domains (school, friends, family and dating).  Acceptability of SCIPT-A from the patient perspective using Client Satisfaction Questionnaire (CSQ-8) and adherence to treatment.  Acceptability of SCIPT-A from the clinician perspective using a clinician treatment satisfaction questionnaire adapted from CSQ-8. | Acceptability of SCIPT-A from the patient perspective: Most parents (N = 23, 86.9%) and adolescents (N = 27, 88.9%) reported being "mostly satisfied" to "very satisfied" with treatment based on CSQ-8.  Acceptability of SCIPT-A from the clinician perspective: Overall positive (social work clinicians N = 4 and medical providers N = 7) based on the CSQ-8. 100% of the social workers and 86% of the medical providers reported that the quality of care provided by SCIPT-A was "good" or "excellent."  15/29 (51.7%) patients improved after 8 weeks of brief IPT-A sessions and did not need medication nor referral to a mental health specialized care setting.   Patients in intervention group experienced a greater decrease in depression symptoms (CDRS-R Cohen's d = 0.35) and improvement in overall severity (CGI-S Cohen's d = 0.84) compared to control group.  Only 7/19 (37%) in the control group were able to receive treatment. At week 16, patients who received treatment experienced better outcomes on the CDRS-R, CGAS, and CGI (p < .05 each).   No significant treatment group differences for primary outcomes. This study was not powered to find statistically significant differences between treatments.   SCIPT-A is an acceptable and potentially beneficial model for providing depression services in primary care for urban Latino adolescents. |
| Clarke 2016 (U.S.)  Cognitive behavioral therapy in primary care for youth declining antidepressants: A randomized trial | Experimental Study, Randomised Controlled Trial. | Warning issued in 2004 by the US Food and Drug Administration regarding the use of antidepressants and suicidality in children. | 212 adolescents 12-18 years old with major depression who declined/discontinued antidepressant medication were recruited between Sept 2006-June 2010. | To evaluate the impact of brief Cognitive Behavioral Therapy (CBT) as an alternative to antidepressant medication. | Control group: Self-selected Treatment As Usual (TAU). Experimental group: TAU plus individual CBT.  Brief CBT program consisted of 2, 4-session modules with 6 visits delivered by therapists with a master's degree and experience in CBT with biweekly supervision. | Primary measure was time to major depression diagnostic recovery defined as ≥ 8 weeks of no/minimal symptoms and no/little impairment.  Secondary measures included depression treatment response defined as â‰¥ 8 weeks below the threshold of 5 or more major depression symptoms required for full diagnosis. | TAU group had an average of 18 weeks until response (95% CI [14.7, 21.3] versus an average of 13.3 weeks for CBT group (95% CI [10.6, 15.9]).  TAU group had an average of 30 weeks to recovery (95% CI [25.3, 34.7]) compared with an average of 22.6 weeks for CBT group (95% CI [18.7, 26.5]. This benefit persisted for at least 1 year after baseline measure.  TAU group reported significantly higher rates of psychiatric hospitalization at 1-year follow-up compared with CBT group (8.5% vs 0.9%, P = .01, odds ratio [95% CI] = 9.7 [1.2, 78.3]).  Brief, primary care CBT can help reduce the risk of future recurrent depression episodes and the use of hospital services in adolescents unreceptive to antidepressant medication. |
| Yucel 2020 (U.S.)  Racial/ethnic differences in treatment quality among youth with primary care provider-initiated versus mental health specialist-initiated care for major depressive disorders. | Correlational, Retrospective Cohort Study. | None specified. | 6950 patients met inclusion/exclusion criteria. | To compare the racial/ethnic differences in treatment quality among adolescents with primary care provider-initiated versus mental health specialist-initiated care for major depression. | Primary Care Providers (PCPs) included family practice/general practice, pediatrics, internal medicine, nurse, and rural health clinic registered nurse.   Social Works/Psychologists (SWPs) included psychologist, licensed professional counselor, licensed master social worker, or case management.  Treatment options for depression included Cognitive Behavioural Therapy (CBT), Interpersonal Psychotherapy (IPT) and antidepressant medications with benchmarks established based on the literature. | Quality of depression services was measured using the minimum adequacy of treatment and mental health (MH)-related hospitalization.  Adequate depression treatment for psychotherapy was a minimum of 4 sessions of either CBT/IPT over 12 weeks.  Adequate treatment for medication was a minimum of 84 days of psychopharmacotherapy during a 120-day treatment period.   Mental health hospitalization identified as any inpatient claim associated with mental disorder diagnosis. | Minority patients received lower quality of care for depression compared to White patients. Hispanics and Blacks were approximately 30% less likely to receive adequate treatment for depression (Hispanics OR: 0.67; 95% CI [0.6, 0.8]) (Blacks OR: 0.66; 95% CI [0.6, 0.8]).  Hispanic patients had the highest risk of having mental health-related hospitalization. They were 50% more likely to undergo mental health-related hospitalization (OR: 1.53; 95% CI [1.1, 2.2]) compared to White patients.   The interaction between race/ethnicity and type of provider who initiated depression services was not statistically significant. Future research is needed to better understand the barriers to treatment completion among minority children. |
| Harder 2019 (U.S.)  Improving Adolescent Depression Screening in Pediatric Primary Care. | Quality Improvement Learning Collaborative. | The U.S. Preventive Services Task Force guidelines recommended in 2009 routine screening for depression in children and adolescents aged 12-18 years in primary care if there's a system in place for treatment and follow-up. The AAP helped develop over the last 10 years educational materials and tools to support this recommendation, but screening rates amongst pediatricians remained low (less than 25%) based on a 2013 AAP survey. | In 2014, 38 pediatric primary care clinics were part of a Quality Improvement (QI) Learning Collaborative through the Child Health Advances Measured in Practice (CHAMP) network created by the Vermont Child Health Improvement Program (VCHIP).  21 were control clinics (n=772 patients) and 17 were participating clinics (n=792 patients). | To increase adolescent depression screening at well-visits to at least 95%, and to increase initial management of patients with positive screening to at least 95%. | Funding provided by the Vermont Department of Health to VCHIP to lead a QI learning collaborative in adolescent depression. The model used for the QI learning collaborative was based on the Institute for Healthcare Improvement Breakthrough Series Collaborative.  Multidisciplinary teams (physicians, nurses, and administrative staff) reviewed practice-specific baseline data, developed clinic-specific goals, tested changes using Plan-Do-Study-Act cycles, and measured improvements every month.   Team training included a day-long learning session and a minimum of 3/6 coaching calls over 7 months. Incentives in the form of training credits were provided to physicians. | Screening rates using a validated screening tool: 16 clinics used the Patient Health Questionnaire (PHQ-9) and 1 clinic used PHQ-2 followed by PHQ-9 for positive results.  Documentation of an initial plan of care for patients who screened positive (PHQ-9 score ≥10), and follow-up actions (e.g., referral to mental health professional, prescribing medication, and follow-up visits).  Mental Health Practice Readiness Inventory (MHPRI) completed at baseline and end of the collaborative to assess organizational support in place (e.g. community resources, financing, clinical information systems and decision support for clinicians). | Monthly Reviews: Average percentage of adolescents screened for depression at the clinic-level increased from 34% to 97% over the 7-month period.  Average percentage of positive depression screens with a documented initial plan of care increased from < 80% to 100% over the 7-month period.  Average clinic MHPRI score improved significantly (12.9%) from the start of the QI collaborative (M= 2.03, SD= 0.29) to after (M= 1.77, SD= 0.44), t(16) =−2.64, P= .02). Specific improvements in 2/5 MHPRI domains (clinical information systems and decision support).  Annual Review: In 2014, adolescents at participating clinics had 3.5 times greater odds (95% CI [1.14, 10.98], P= .03) of being screened for depression and 37.5 times greater odds (95% CI [7.67, 183.48] P<.0005) of being screened with a validated tool than adolescents at control clinics.  No difference between participating and controls clinics in the odds of having an initial plan of care documented, but having Medicaid and being screened for depression in 2012 predicted having an initial plan of care in 2014.   These results could be explained by the fact that documentation was an improvement at the clinician-level versus screening was an improvement at the clinic-level. More research needed on initial plans of care and treatment outcomes. |
| Shippee 2018 (U.S.)  Effectiveness in Regular Practice of Collaborative Care for Depression Among Adolescents: A Retrospective Cohort Study. | Correlational, Retrospective Cohort Study. | The U.S. Preventive Services Task Force guidelines recommended screening for adolescent depression if there's a system in place for treatment and follow-up.  The National Network of Child Psychiatry Access Programs (NNCPAP) provide mental health services, but these services vary depending on location and are not integrated into every practice. | 162 EMERALD patients and 499 similarly eligible non-EMERALD patients (12-18 years-old) at a large multi-specialty practice in the U.S. Midwest. | To evaluate the effectiveness of EMERALD (Early Management and Evidence-based Recognition of Adolescents Living with Depression). | EMERALD (Early Management and Evidence-based Recognition of Adolescents Living with Depression) was based on a collaborative care model for adults. It was a quality improvement project that became standard practice.   PHQ-9A was routinely administered in well-child visits for patients ages 12-17. A primary care clinician made the initial diagnosis of depression in consultation with a child/adolescent psychiatrist if needed.   Patients were referred to a registered nurse care coordinator trained in depression management, including developing a relapse prevention plan. Treatment included primarily cognitive behavioural therapy. | 9-item Patient Health Questionnaire Modified for Adolescents (PHQ-9A) to screen for symptoms of depression.  Primary discharge criterion was sustained depression remission (PHQ-9A score <5 for 3 consecutive months)  6-month remission of depression (score <5).  6-month treatment response ( >50% reduction from baseline). | EMERALD group had treatment response rates of 43%-44% versus 28%-30% for comparison group. Remission rates were 29%-31% for EMERALD group versus 19%-20% for comparison group.   EMERALD patients had better adjusted rates of depression remission (11 percentage points higher, p=.035) and treatment response (14 percentage points higher, p<.001) than comparison group.  Collaborative care for adolescent depression was associated with higher rates of improved mood after 6 months, compared with usual care. |
| Rapp 2017 (U.S.)  Integrated Primary Medical-Behavioral Health Care for Adolescent and Young Adult Depression: Predictors of Service Use in the Youth Partners in Care Trial. | Experimental Study, Randomised Controlled Trial. | Integrating mental health services in primary care had been incentivized in U.S. health system because of its potential to achieve the triple aim of improving health, the quality and experience of care, and reducing costs. | 344 patients with major depression or dysthymia from 6 primary care clinics affiliated with 5 health organizations (managed care, public sector and academic medical centres). Over half of the sample spoke a language other than English at home of which 89.6% were Spanish speakers. | To evaluate a collaborative care model for adolescent depression services in primary care. | The Youth Partners in Care (YPIC) was developed to evaluate access to evidence-based depression services for patients 13-21 years old using a collaborative care model in primary care.   Intervention Group: The model included leaders who adapted and implemented the intervention and care managers who provided expertise to primary care clinicians and Cognitive Behavioral Therapy (CBT). Treatment was available in English/Spanish.  Control Group: Usual care involved referral for mental health care services with medication management/psychotherapy in primary care. | Major Depression and dysthymia diagnosis using the Mood Disorders module of WHO CIDI-12.  Mental Health Treatment: Receipt of mental health services in the past 6 months, derived from patient response on the Service Assessment for Children and Adolescents (SACA). | Significantly higher treatment rates in the intervention group compared to the control group, including the following significant moderators:   (1) Increasing age (13-21 years old) was associated with lower treatment rates in the control group (p<.0001). Age-related decline in service use is noted in older adolescents.  (2) Treatment rates were higher in the intervention group when English was the primary language spoken at home, but no differences if another language was spoken (p=.023). Lower-service use is noted in lower levels of acculturation in the United States. |
| Ellis 2019 (Australia)  Assessing the quality of care for paediatric depression and anxiety in Australia: A population-based sample survey. | Correlational, Cross-Sectional Study. | Australia developed several initiatives to address mental health services at a national level, including the National Mental Health Strategy (1992), 5-year mental health plan (1993-2014), a National Action Plan of Mental Health under the Council of Australian Governments (2006-2012), the Better Access initiative (2006) and annual National Report Cards on Mental Health and Suicide Prevention (2012). | 133 medical records for depression, and 267 medical records for anxiety for patients ≤ 15 years receiving care in 2012-2013 from hospitals/emergency department (1-3%), General Practices (GPs) (86% depression, 58% anxiety), and paediatrician’s offices (12% depression, 41% anxiety). | To evaluate adherence to depression and anxiety Clinical Practice Guidelines (CPGs) from a previous study, the Care Track Kids (CTK), that assessed the quality of health services for children in Australia in inpatient and ambulatory health settings. | 28 medical record audit indicator questions (15 for depression and 13 for anxiety) were identified through a review of the literature and expert consultation on CPGs. | Diagnosis of anxiety/depression using ICD-10 AM code (or equivalent) in the medical records.  Adherence to CPGs was measured by number of indicators scored as "Yes". For depression, Bundle A covered assessment (children/adolescents who presented with suspected depression received appropriate assessment) and Bundle B covered management (children/adolescents with depression received appropriate information and treatment support and management). | Bundles: The lowest compliance was found for GPs compared to other health settings with low adherence to assessment = 30.3% (95% CI [11.7, 55.3] and management bundles for depression = 31.8% (95% CI [7.7-66.5]).  Indicators: For GPs, compliance was the lowest for ensuring children with depression had an emergency safety plan = 41% (95% CI [10.5, 78.4]). Highest compliance was for medications (not prescribing antidepressants as a first-line intervention) = 90.5% (95% CI [68.6, 99.0].  Overall, lower levels of compliance were reported for GPs for anxiety and depression than other health settings. Recommendations to use these baseline results as benchmarks, provide training, and integrate prompts in electronic health records as reminders. |
| Richardson 2014 (U.S.)  Collaborative care for adolescents with depression in primary care: a randomized clinical trial. | Experimental Study, Randomised Controlled Trial. | The U.S. Preventive Services Task Force guidelines recommended routine screening for depression in adolescents. | 101 patients (aged 13-17 years) recruited from 9 pediatric and family medicine clinics in the Group Health system between April 2010 and March 2011. 50 in intervention group and 51 in control group.   Clinics were located in 3 urban areas in Washington State, and selected for their greater patient diversity and higher number of adolescent patients. | To evaluate whether a collaborative care model for adolescents with depression improves depression compared to usual care. | The Reaching Out to Adolescents in Distress (ROAD) is a 12-month intervention that included an initial in-person session and regular follow-ups with clinicians (every 1-2 weeks). Treatment options consisted of brief Cognitive Behavioural Therapy (CBT), antidepressant medication, and/or both. Two 4-session modules of brief CBT were delivered by trained depression care managers (clinicians hired for the study).  The control group received usual care which included receiving depression screening results and accessing mental health services through Group Health. | Primary: Change in symptoms of depression on a modified version of the Child Depression Rating Scale–Revised (CDRS-R) from baseline to 12 months.   Secondary: Change in Columbia Impairment Scale score (CIS), depression response (≥50% decrease on the CDRS-R), and remission (PHQ-9 score <5). | High adherence to treatment. 86% of patients in intervention group met quality standards for medications/psychotherapy compared with only 32% in the Youth Partners in Care Study (another similar study). Differences included duration (12 months), involvement of parents, and active outreach efforts.  Decreases in CDRS-R scores were higher after 12 months in intervention group with mean score of 27.5 (95%CI [23.8, 31.1]) compared with 34.6 (95% CI [30.6, 38.6]) in control group.  Both intervention and control groups experienced improvement in CIS scores with no significant differences between groups.   At 12 months, intervention group was more likely than control group to achieve depression response (67.6% vs 38.6%, OR = 3.3, 95% CI [1.4, 8.2] P = .009) and remission (50.4% vs 20.7%, OR = 3.9, 95% CI [1.5, 10.6] P = .007).  This collaborative care model resulted in greater improvement in depressive symptoms after 12 months compared to usual care. Screening is not enough to result in increases in treatment rates, a strategy is needed to engage patients with support from their parents. |
| Rinke 2019 (U.S.)  Effect of Mental Health Screening and Integrated Mental Health on Adolescent Depression-Coded Visits. | Correlational, Retrospective Cohort Study. | None specified. | 42,165 patients (11-23 years old) from 19 urban pediatric and family medicine primary care sites affiliated with a tertiary care medical center. | To evaluate if screening and integrating mental health clinicians affect adolescent depression's coded visits in primary care. | (1) Baseline: No standard screening protocol. Access to generalist social workers.  (2) Mental health screening: Screening at health care maintenance visits using the Pediatric Symptom Checklist 17 (PSC-17) for 4- to 11-year-olds, and the Youth PSC-17 (Y-PSC-17) for 12-year-olds and older.   If patients screened positive, primary care clinicians administered more comprehensive depression diagnosis tools and/or referred patients to mental health clinicians for diagnosis.  (3) Integrated mental health services: Evidence-based treatments delivered by pediatric psychologists and psychiatrists (medications). | Change in the percent of adolescent depression/subthreshold depression-coded visits between the baseline period and 1 of 2 intervention periods.  Comparing practices with and without the mental health screening intervention or integrated mental health clinicians. | Depression-coded visits increased significantly more in clinics that completed mental health screening (ratio of odds ratios = 1.22; 95% CI [1.00, 1.49]) and clinics with integrated mental health clinicians (ratio of odds ratios = 1.58; 95% CI [1.30, 1.93]).   Significant increase in percentage of depression-coded visits in clinics with integrated mental health clinicians compared to clinics with mental health screening only (ratio of odds ratios 2.03 (95% CI [1.58, 2.59] P< .001).  Adolescent mental health screening and integrated mental health practitioners increased depression-coded visits in primary care. Percentage of diagnosed patients was low (2.8%) compared to the incidence of at least 10% with a prevalence of 20% throughout adolescence, using depression-specific assessment tools like the Patient Health Questionnaire may help improve these results in the future. |
| Forman-Hoffman 2016  Screening for Major Depressive Disorder in Children and Adolescents: A Systematic Review for the U.S. Preventive Services Task Force. | Systematic Review. | The U.S. Preventive Services Task Force guidelines recommended in 2009 routine screening for depression in children and adolescents aged 12-18 years in primary care. | Types of studies included randomized and non-randomized trials, recent systematic reviews, test-retest studies, and large cohort studies. Studies had to have at least 50% of participants with a diagnosis of major depression with screening completed in primary care. | To update the 2009 U.S. Preventive Services Task Force systematic review on screening for and treatment of major depression in children and adolescents in primary care. | Not applicable. | Not applicable. | Limited evidence from 5 studies showed that the Beck Depression Inventory and Patient Health Questionnaire for Adolescents were accurate at identifying adolescents with major depression in primary care.   6 studies evaluated treatment options including fluoxetine, combined fluoxetine and cognitive behavioral therapy, escitalopram, and collaborative care. Results showed benefits of treatment among adolescents, with no associated harms.  More research needed, specifically for populations younger than 12 years old. Challenges with conducting studies in child and youth mental health may explain the limited evidence available (e.g. lower recruitment rates, limited funding available, and higher attrition rates). |
| Radovic 2014 (U.S.)  Primary care providers' initial treatment decisions and antidepressant prescribing for adolescent depression. | Correlational, Cross-Sectional Study. | National Guidelines for Adolescent Depression in Primary Care recommended Cognitive Behavioral Therapy (CBT) and/or Selective Serotonin Reuptake Inhibitors (SSRIs) for moderate or severe depression. | 57 pediatric primary care clinicians working within an integrated behavioral health network in the greater Pittsburgh, Pennsylvania area. Clinicians included pediatricians, pediatric nurse practitioners and physician assistants. | To understand how pediatric primary care clinicians decide to treat adolescents with depression. | Pediatric primary care clinicians screen adolescents 15-17 years old at well-child visits (Patient Health Questionnaire-9 Modified), have access to mental health therapists onsite/nearby, a senior mental health therapist for supervision and a shared electronic health record. Average time to seeing new patients is 7 days.  Training provided to primary care clinicians on how to evaluate and manage common mental health conditions including depression within the last year. | 93-item survey completed anonymously that collected information on beliefs about antidepressants and suicide risk, depression knowledge, psychosocial beliefs, treatment decision-making, factors influencing treatment decisions, and perceived barriers. | Only a third of 58 clinicians recommended an antidepressant (25% for moderate, 32% for severe).  Severity of depression didn't increase the chances of prescribing an antidepressant (OR 1.58 [95% CI 0.80,3.11] p=.19).   Antidepressants were more likely to be recommended with greater knowledge of depression (OR 1.72 [95% CI 1.14, 2.59] p=.009) and access to an onsite mental health therapist (OR 5.13[95% CI 1.24, 21.2] p=.02). Less likely to be recommended by clinicians who reported higher burden with psychosocial concerns (OR 0.85 [95%CI 0.75, 0.98] p=.02).  Factors that played a role in treatment decisions included knowledge of depression and treatments, level of comfort with handling psychosocial problems, and availability of a mental health therapist in the primary care clinic. |
| Dickerson 2018 (U.S.)  Cost-effectiveness of Cognitive Behavioral Therapy for Depressed Youth Declining Antidepressants. | Experimental Study, Randomised Controlled Trial. | None specified. | 212 adolescents 12-18 years old with major depression who declined/discontinued antidepressant medication were recruited between Sept 2006-June 2010. | To evaluate the cost-effectiveness of a brief Cognitive Behavioral Therapy (CBT) as an alternative to antidepressant medication. | Control group: Self-selected Treatment As Usual (TAU). Experimental group: TAU plus individual CBT.  Brief CBT program consisted of 2, 4-session modules with 6 visits delivered by therapists with a master's degree and experience in CBT with biweekly supervision. | Children’s Depression Rating Scale-Revised (CDRS-R) to measure symptoms of depression.  Depression Free Days (DFDs) calculated based on CDRS-R scores at 12- and 24- month follow-up points.   Quality-Adjusted Life-Years (QALYs) where DFDs (full health) were assigned the weight of 1.0 and depressive episodes the weight of 0.6 based on empirical studies.   Costs calculated at 12- and 24-month follow-up points adjusted to 2008 US dollars. This included intervention service costs, usual care costs, and family costs. | CBT group had 26.8 more DFDs (P = .044) and 0.067 more QALYs (P = .044) on average compared with TAU group at the end of the 1-year follow-up.   Total costs were $4,976 less (P = .025) in CBT group than TAU group by the end of the 2-year follow-up.  Brief primary care CBT was more cost-effective compared to TAU at the end of the 2-year follow-up for adolescents unreceptive to antidepressant medication. |
| Leslie 2018 (U.S.)  Patient-Administered Screening Tool May Improve Detection and Diagnosis of Depression Among Adolescents. | Quality Improvement Project. | Recommendation issued in 2016 by the American Academy of Pediatrics to screen for depression every year starting at age 11.  No existing system in place at the selected primary care pediatric clinic to screen for depression. | One primary care pediatric clinic in rural South Carolina with 1/5 clinicians participating in the Quality Improvement (QI) project. Pre-intervention data included 282 patients and post-intervention data included 88 patients. | To evaluate if a self-administered adolescent depression screen would improve the detection, diagnosis, and management of major depression in a pediatric primary care clinic. | Training provided on the purpose, administration, and interpretation of the Patient Health Questionnaire for Adolescents (PHQ-9a).  Adolescents between the ages of 11-21 years old completed the PHQ-9a in a private exam room with or without the presence of family member/caregiver.   Clinician reviewed the PHQ-9a score and managed depressive symptoms as appropriate. Scores > 9 signal moderate depressive symptoms needing more in-depth evaluation by the clinician with either antidepressant treatment and/or external referral for treatment. | Number of PHQ-9a completed.  Number of adolescents with scores > 9 on the PHQ-9a.  Number of adolescents with a diagnosis of depression during visits to the clinic.  Number of adolescents prescribed a new antidepressant medication or referred externally for treatment. | PHQ-9a increased depression diagnosis. 5% (15/282) of the adolescents were diagnosed with depression in the pre-intervention period versus 17% (15/88) in the post-intervention period. Compliance rate for administering the depression screens was 88% (77/88).   Adolescents presenting for sick visits were more likely to show symptoms of depression compared to those presenting for well visits. Of the 77 patients screened, 43% (33/77) were performed during well visits and 57% (44/77) at sick visits. 3% (1/33) scored > 9 on the PHQ-9a during well visits and 16% (7/44) during sick visits.  A patient-administered depression screening tool administered to adolescents during well/sick visits helps improve the identification and diagnosis of depression. It's recommended not to limit screening to annual well visits. |
| McCann 2012 (Australia)  Young people with depression and their satisfaction with the quality of care they receive from a primary care youth mental health service: a qualitative study. | Exploratory, Qualitative Study. | The Australian head-space model of primary care service for people 12-25 years old was developed in 2006 and funded by the Common-wealth Government to provide primary care services including mental health. | 26 patients with a primary diagnosis of depression between 16-22 years old recruited from the same primary care youth mental health service. Median duration of treatment was 4-5 months. | To evaluate patient satisfaction with depression services received in primary care. | Depression services are provided by clinicians in a primary care youth mental health service identified as a headspace centre. Clinicians included mental health nurses, psychologists, social workers, occupational therapists and psychiatrists. | Sample interview questions to evaluate patient satisfaction with depression services:  What do mental health professionals in this service do, if anything, to help you with depression?   What do mental health professionals in this service do, if anything, to make it difficult for you with depression?  What could mental health professionals in this service do, if anything, to help you with depression? | Themes linked to patient satisfaction: (1) Clinicians being youth-friendly: understanding, respectful, honest, unbiased, proactive in ensuring continuity of appointments, and opportunity to change clinicians if needed (absence/break-down of engagement). (2) Clinicians adopting a broad-approach to care: psychosocial therapies, occasional use of antidepressants, and including primary parent/carer. (3) Care facilitating recovery: ongoing therapeutic dialogue between the patient and clinician to resolve problems and cope with situations. |
| Farley 2020 (U.S.)  Identification and Management of Adolescent Depression in a Large Pediatric Care Network. | Correlational, Cross-Sectional Study. | The American Academy of Pediatrics (Guidelines for the Management of Adolescent Depression in Primary Care) and US Preventive Services Task Force have developed clinical practice guidelines to support appropriate care, including routine depression screening for adolescents aged 12 years and older. | All patients who attended their age 16 well-visits between Jan 2014-Aug 2016 at 1 of the organization's 31 primary care clinics (n=10,713). The organization is a large pediatric care network in the US Mid-Atlantic Region. | To evaluate the screening, initial management, and follow-up of adolescent depression in primary care. | Automated electronic screening system for 5 types of mental disorders including depression.  Screening for adolescent depression started at age 16 during well-visits (a higher number of patients expected at this age for their well-visits due to vaccination and driver's permit documentation requirements). The age requirement was changed to 12 in line with national guidelines post-study.  Follow-up actions documented in Electronic Health Record (EHR) using a drop-down menu of options with a 1-year follow-up for patients with elevated scores.  Clinicians received in-person/electronic information about depression, screening, the Patient Health Questionnaire-Modified for Teens (PHQ-9-M), how to review and follow-up on the results, discuss results with families, available treatment resources and legal information. | PHQ-9-M completed by patients to measure depressive symptoms and severity.  Follow-up actions for patients with elevated PHQ-9-M scores documented in EHR (suicide risk/emergency procedures, primary management in clinic, referred to behavioural health provider, already receiving services, or no action needed/error).  1-year follow-up for patients with elevated PHQ-9-M scores in EHR. | High screening rate, 76.3% (n=6,981) of patients attending their age 16 well-visits were screened with PHQ-9-M. About 25% of patients had an elevated score (19.2% mild n=1,331, and 6.7% moderate-to-severe n=466).  High follow-up rates, 81.5% of patients with elevated scores (n=1,471) had follow-up actions documented in the EHR with most frequent option was referral to behavioural health clinicians (34.33%, n=160).  1-year follow-up rates varied depending on level of severity of symptoms, 75.4% of patients with moderate-to-severe scores (n=349) and 39.9% of patients with mild scores (n=530) had EHR-documented follow-up within 1 year.   Integrating routine screening practices facilitates follow-up actions based on the results. Recommendations to review PHQ-9-M items with the patient to validate the results, develop clearer policies on EHR documentation for consistency ("problem list" documentation), and set a process for sharing elevated scores/results with other specialists. |
| Thompson 2019 (U.S.)  Collaborative Care for Depression of Adults and Adolescents: Measuring the Effectiveness of Screening and Treatment Uptake. | Correlational, Retrospective Cohort Study. | The U.S. Preventive Services Task Force recommended in 2015 routine screening for adult depression in primary care. | 25,369 patients (12-25 years old) from 10 primary care clinics in Chicago. | To evaluate the effectiveness of a collaborative care for depression across primary care clinics. | Second year implementation of a collaborative care model in a large academic medical center that includes: setting-up an algorithm in the EHR to determine eligibility for screening (≥ 12 years old and no assessment within the last 12 months), screening eligible patients initially with PHQ-2 then PHQ-9, referring patient using EHR prompts based on results, contacting patient within 7 days to discuss symptoms and treatment options beyond medication, and following-up in 6-8 weeks. | Proportion of patients screened initially with PHQ-2 (Patient Health Questionnaire) then the PHQ-9.  Proportion of patients referred and treated. | Having an integrated EHR screening system increased screening rates. 79% (n=20,053) of eligible patients were screened with PHQ-2 compared to only 7.18% (n=32,495) screened with PHQ-2 within the 12 months pre-intervention.  Adolescent age (12-17 years old) was associated with disproportionate PHQ-9 screenings and with treatment disengagement. Only 11.35% of referred patients received treatment (n=42/137). More research needed on patient perspectives on how to improve treatment uptake, specifically with adolescent patients and patients of color. |
| Martínez 2018 (Chile)  Remote Collaborative Depression Care Program for Adolescents in Araucanía Region, Chile: Randomized Controlled Trial. | Experimental Study, Randomised Controlled Trial. | The Chilean Ministry of Health issued recommendations for treating depression in adolescents in clinical practice.  Chile developed an evidence-based depression intervention with plans to scale in resource-constrained settings. However, there were barriers at the primary care-level to treating depression with an uneven geographical distribution of mental health resources, specifically for low-income people. | 16 primary care clinics in the Araucanía Region. 143 adolescents 13-19 years-old with major depression (intervention group n=65, and control group n=78). | To evaluate the impact of a Remote Collaborative Depression Care (RCDC) intervention for adolescents with major depression. | Web-based training provided to clinics from both groups in clinical guidelines for the treatment of adolescent depression. This program was based on recommendations from the Chilean Ministry of Health.  Clinics in RCDC program had access to 3 months of remote supervision by psychiatrists: (1) Shared EHR to facilitate communication between clinicians in the primary care clinics and clinicians with specialized expertise in child and adolescent mental health (University of Chile, Faculty of Medicine). This was used as a discussion forum to provide real-time support during the diagnosis and treatment process. (2) Phone patient monitoring calls at 1, 2, 3, 6, and 9 weeks post baseline assessment per adolescent and primary caregiver.  Control group received enhanced usual care where clinicians had access to the baseline diagnostic assessment results and were encouraged to follow the recommendations of the Ministry of Health of Chile for the management of adolescent depression. | Semi-structured clinical interview MINI-KID (Mini-International Neuropsychiatric Interview For Children and Adolescent) was used to determine eligibility and diagnose children and adolescents with major depression.  Primary measure was the effectiveness of the RCDC program assessed with mean Beck Depression Inventory scores evaluated at baseline and after 12 weeks.  Secondary measure was change in health-related quality of life (HRQoL) evaluated at baseline and after 12 weeks using the KIDSCREEN questionnaire (frequency/intensity of behaviors, feelings, or attitudes within the last week).   Patient adherence and satisfaction with RCDC treatment, and clinician satisfaction were measured after 12 weeks using self-reported questionnaires (with a brief interview for pharmacological adherence). | Only a third of the patients took their medications as prescribed (n=44). No significant differences between groups after 12 weeks. No significant differences in depressive symptoms or HRQoL between groups after 12 weeks. This may be explained by a high turnover rate of primary care clinicians in remote areas and difficulties training new clinicians.  Overall primary care clinicians found RCDC helpful in their work but shared that the online platform needed to be more user-friendly with dedicated time to use it, and shared preference to collaborating with specialists in-person.  Significant difference in patient satisfaction with psychological care where the intervention group scored higher than the control group (Wilcoxon rank-sum test P=.04). This may be explained by the presence of more stable personnel as part of the care team (psychologists). It is recommended to explore the role of psychologists as case managers in future research and provide more training in psychoteraphy as the treatment focused on medications.  Satisfaction with psychological care, in both groups, was related to a significant change in depressive symptoms after 12 weeks (beta=−4.3, 95% CI [−7.2,−1.3]). |
| Bose 2021 (U.S.)  Implementation of Universal  Adolescent Depression  Screening: Quality  Improvement Outcomes | Quality Improvement Project | The American Academy of Pediatrics (Guidelines for the Management of Adolescent Depression in Primary Care) and US Preventive Services Task Force have developed clinical practice guidelines to support appropriate care, including routine depression screening for adolescents aged 12 years and older. | 184 adolescents (12-18 years old) who visited the privately-owned pediatric primary care clinic in the Midwest for annual wellness checks in 2018-2019 (86 pre-implementation and 98 post-implementation). | To support early identification of adolescent depression by implementing universal  screening during  annual wellness checks in accordance with national guidelines. | A screening protocol was developed to provide guidance to clinicians on how to implement adolescent depression screening.  Clinicians documented the  score for each patient in their electronic health record.  Symptom management was based on the Guidelines for Adolescent Depression  in Primary Care (no intervention needed, continue to monitor and rescreen, refer for counseling, initiate pharmaceutical treatment, or refer to behavioral health for  counseling, psychotherapy, and/or pharmacological management). | Patient Health Questionnaire-9 modified for Adolescents  (PHQ-A). | During the two-month implementation period, 74.5% (73/98) adolescents received a documented depression  screen compared to 0% during the same two-month period from the previous year.  The rate of new depression diagnoses, mental health referrals, and initiation of pharmaceutical treatment in the pre/post-implementation samples increased to 13.3%, 9.2%, and 6.1%, respectively. Using an exact Pearson chi-square test at the 0.05 level,  there was evidence of a pre/post difference in the percentage  of new depression diagnoses (p = .0017) and a pre/post difference in the percentage of mental health referrals (p = .0207). There was no evidence of a pre/post difference in the percentage of pharmaceutical treatment initiation. |
| Holcomb 2021 (U.S.)  Beyond Initial Screening: One-Year Follow-up of Adolescents  with Internalizing Problems on the Pediatric Symptom  Checklist | Correlational, Retrospective Cohort Study. | The American Academy of Pediatrics (Guidelines for the Management of Adolescent Depression in Primary Care) and US Preventive Services Task Force have developed clinical practice guidelines to support appropriate care, including routine depression screening for adolescents aged 12 years and older. | 604 adolescents (12-17.9 years old) who visited the pediatric primary care clinic, Revere  Healthcare Center (RHC) in Massachusetts, for annual wellness checks between January 1, 2017, and December 31, 2018. | To evaluate screening outcomes for adolescent depression in a mixed socioeconomic status pediatric primary care clinic. | The clinic is part of a  network of 9 practices affiliated with Massachusetts  General Hospital (MGH) requiring annual screening for adolescent depression and providing behavioural health services. | 17-item Pediatric Symptom Checklist’s internalizing subscale (PSC-17P-INT) | 93.9% of adolescents (567/604) completed the PSC-17P-INT at both visits in 2017 and 2018.  Patients who scored positively on their first PSC-17P-INT were about 9 times more likely to receive subsequent behavioural health services than patients who screened negative (24.3% vs 2.6%, χ2=59.65, p < 0.001).  Of the 70 patients who were at risk on the PSC-17P-INT at their first screen,  almost two-thirds (44, 62.9%) remained at risk at their second screen (χ2=143.60, p < 0.001). |
