# Supplementary material for: Achieving Quality Integrated Care for Adolescent Depression: A Scoping Review
Source: J Prim Care Community Health. 2022 Nov 7;13:21501319221131684. doi: 10.1177/21501319221131684 (PMC9647275; doi:10.1177/21501319221131684)
Supplement: sj-docx-3-jpc-10.1177_21501319221131684 – Supplemental material for Achieving Quality Integrated Care for Adolescent Depression: A Scoping Review [file sj-docx-3-jpc-10.1177_21501319221131684.docx]

Supplement 3. Definitions of Integrated Care from Included Studies

| **Author/Year/Title/**  **Country** | **Definition** |
| --- | --- |
| Wright 2016 (US)  The Costs and Cost-effectiveness of Collaborative Care for Adolescents With Depression in Primary Care Settings: A Randomized Clinical Trial. | Collaborative Care: “Patient engagement strategies, patient choice of treatment, provision of evidence-based treatments in the primary care setting, active follow-up by a depression care manager (DCM) to assist with treatment adherence and stepped-care approaches to adjust treatment based on patient treatment response." |
| Buchanan 2020 (US)  Integration of behavioral health services and adolescent depression screening in primary care. | Integrated Behavioural Health: “A dynamic process whereby behavioral health clinicians (e.g., psychologists, psychiatrists, clinical social workers) actively engage with patients in the clinic on a flexible, as-needed schedule through the pediatric primary care provider's request, either immediately or through follow-up.” |
| Mufson 2018 (US)  Stepped care interpersonal psychotherapy treatment for depressed adolescents: A pilot study in pediatric clinics. | Collaborative Care: "A greater role for nonmedical specialists to augment primary care's management of mental health issues. This model often includes (1) enhanced patient education, (2) use of allied health professionals to provide closer monitoring of outcomes, adverse effects and adherence to treatment, (3) use of standardized measures to track outcomes, (4) a psychiatrist to provide supervision of the care managers regarding changes in medication, (5) stepped care approaches that increase intensity of treatment for patients who do not respond to initial lower intensity approach or whose symptoms persist, and (6) care managers who may also provide the option of brief evidence-based psychotherapy." |
| Shippee 2018 (US)  Effectiveness in Regular Practice of Collaborative Care for Depression Among Adolescents: A Retrospective Cohort Study. | Collaborative Care: "Four essential elements: team driven (incorporating primary care providers, care managers, and psychiatric specialists), population focused (employing registries and systematic screening), measurement guided (emphasizing patient-reported outcomes) and evidence based." |
| Rapp 2017 (US)  Integrated Primary Medical-Behavioral Health Care for Adolescent and Young Adult Depression: Predictors of Service Use in the Youth Partners in Care Trial. | Integrated Primary Medical and Behavioural Health Care: "The inclusion of behavioral health services as part of primary care using tightly integrated on-site teamwork." |
| Rinke 2019 (US)  Effect of Mental Health Screening and Integrated Mental Health on Adolescent Depression-Coded Visits. | Integrated mental health services: "True integration increases primary care provider knowledge about mental health diagnoses, and comfort in asking patients questions about mental health diagnoses, leading to increased rates of adolescent depression diagnoses." |
| Thompson 2019 (US)  Collaborative Care for Depression of Adults and Adolescents: Measuring the Effectiveness of Screening and Treatment Uptake. | Collaborative Care: “Population-based approaches, such as collaborative care models (CCM), offer unique opportunities to reduce biases of individual-level screening by leveraging algorithms and, in this adaptation, the electronic health record (EHR) infrastructure, prompting providers to screen all eligible patients. In addition, CCM interventions optimize limited mental health resources and expand the reach of mental health care into primary care.” |
| Martínez 2018 (Chile)  Remote Collaborative Depression Care Program for Adolescents in Araucanía Region, Chile: Randomized Controlled Trial. | Collaborative Care: “Team-driven, population-focused, measurement-guided, and evidence-based provision of care.” |
